# Growth differentiation factor 7 autocrine signaling promotes hepatic progenitor cell expansion in liver fibrosis

**DOI:** 10.1186/s13287-023-03493-3

**Published:** 2023-10-05

**Authors:** Defu Kong, Apostolos Mourtzinos, Janette Heegsma, Hans Blokzijl, Vincent E. de Meijer, Klaas Nico Faber

**Affiliations:** 1 Department of Gastroenterology and Hepatology, University of Groningen, University Medical Center Groningen, Hanzeplein 1, 9713 GZ Groningen, The Netherlands; 2 Department of Surgery, Division of Hepato-Pancreato-Biliary Surgery and Liver Transplantation, University Medical Center Groningen, University of Groningen, Groningen, The Netherlands

**Keywords:** GDF7, Liver fibrosis, LGR5, Progenitor cell, Organoids

## Abstract

**Background and aim:**

Liver fibrosis is prevalent among chronic diseases of the liver and represents a major health burden worldwide. Growth differentiation factor 7 (GDF7), a member of the TGFβ protein superfamily, has been recently investigated for its role in repair of injured organs, but its role in chronic liver diseases remains unclear. Here, we examined hepatic GDF7 expression and its association with development and progression of human liver fibrosis. Moreover, we determined the source and target cells of GDF7 in the human liver.

**Methods:**

GDF7 expression was analyzed in fibrotic and healthy human liver tissues by immunohistochemistry and qPCR. Cell-specific accumulation of GDF7 was examined by immunofluorescence through co-staining of cell type-specific markers on formalin-fixed paraffin-embedded human liver tissues. Public single cell RNA sequence databases were analyzed for cell type-specific expression of *GDF7*. In vitro, human liver organoids and LX-2 hepatic stellate cells (LX-2) were treated with recombinant human GDF7. Human liver organoids were co-cultured with activated LX-2 cells to induce an autocrine signaling circuit of GDF7 in liver organoids.

**Results:**

GDF7 protein levels were elevated in fibrotic liver tissue, mainly detected in hepatocytes and cholangiocytes. In line, *GDF7* mRNA was mainly detected in liver parenchymal cells. Expressions of *BMPR1A* and *BMPR2*, encoding GDF7 receptors, were readily detected in hepatocytes, cholangiocytes and stellate cells in vivo and in vitro. In vitro, recombinant GDF7 promoted liver organoid growth and enhanced expression of the progenitor cell markers (LGR5, AXIN2), but failed to activate LX-2 cells. Still, activated LX-2 cells induced GDF7 and LGR5 expression in co-cultured human liver organoids.

**Conclusions:**

Collectively, this study reveals a role of GDF7 in liver fibrosis and suggests a potential pro-regenerative function that can be utilized for amelioration of hepatic fibrosis caused by chronic liver disease.

**Supplementary Information:**

The online version contains supplementary material available at 10.1186/s13287-023-03493-3.

## Introduction

Liver fibrosis is characteristic to most chronic liver diseases, including alcoholic hepatitis, autoimmune liver disease and non-alcoholic steatohepatitis, among others. Advanced fibrosis can lead to cirrhosis, liver failure and hepatocellular carcinoma, which is associated with increased morbidity and mortality. So far, there are no approved therapies to treat fibrosis, e.g. conventional anti-fibrotic drugs or elimination of potential causes fail to alleviate fibrosis in most patients. At end-stage cirrhosis, liver transplantation is the only option to increase patient survival [1].

Progression of liver fibrosis is orchestrated by an interplay of basically all different cell types in the liver, including the parenchymal hepatocytes and cholangiocytes and the non-parenchymal endothelial cells, Kupffer cells and hepatic stellate cells (HSC), as well as other infiltrating immune cells [2, 3]. A pivotal feature of liver fibrosis pathogenesis is the activation of HSC. HSC constitute 5–8% of all the cells in the healthy liver and are located in the space of Disse between hepatocytes and the sinusoidal endothelial cells [4]. HSC activation is promoted by the interaction with other liver resident cells and infiltrating cells during acute or chronic liver injury caused by hepatic viruses, drugs or metabolic disorders. In response to injury, hepatocytes and Kupffer cells produce various types of cytokines, which include multiple inflammatory and regenerative factors, such as and interleukins [5, 6] and transforming growth factor-β (TGFβ) [7], which act as autocrine or paracrine signaling molecules [2].

The TGFβ family of cytokines includes multiple bone morphogenetic proteins (BMP)/growth differentiation factors (GDF) [8]. To date, about 20 different BMPs/GDFs have been identified and are grouped into subfamilies according to structural similarities. BMPs/GDFs are secreted glycoproteins that control a large array of biological activities including cell proliferation, differentiation and survival that aid in wound healing, embryological tissue development and tissue maintenance [9–11]. TGFβ type cytokines play different roles in chronic liver diseases, including a profibrotic role by activating hepatic stellate cells and anti-inflammatory roles by regulating macrophage plasticity [12]. GDF7, also called BMP12, is well-known for its role in the repair and regeneration of tendon and ligament by regulating stem cell proliferation and differentiation [13]. Accordingly, Zhou et al. demonstrated that GDF7 promotes human adipose-derived stem cell differentiation in vitro [14]. In addition, Dong et al. found that GDF7 ameliorates sepsis-induced acute lung injury by regulating the STING/AMPK pathway [15]. Interestingly, *GDF7* mRNA levels in blood cells are elevated in patients with liver cirrhosis compared to healthy controls [16]. However, the relevance of GDF7 in the pathophysiology of chronic liver disease and its potential role herein remain to be explored.

Thus, in this study, we investigated the expression of GDF7 in healthy and fibrotic human liver tissues to a cell-type specific level. Mechanisms that induce GDF7 expression and its downstream effects were analyzed by cocultures of HSC-LX2 cells and human adult-progenitor cell-derived organoids, as well as direct exposure of these cells to recombinant GDF7.

## Material and methods

### Patients’ samples

The liver specimens for analysis were obtained according to Dutch legislation and the Code of Conduct for responsibly dealing with human-derived material in the context of health research. Control tissue was obtained from human livers that were rejected for a transplant procedure, while fibrotic or cirrhotic liver tissue was obtained from diseased explant livers. Liver tissue was snap-frozen for Q-PCR analysis and formalin-fixed paraffin-embedded for histochemical analysis.

### Human cell culture and treatments

All cells were grown in 5% CO_2_ and 37 °C in ambient air. Human liver organoids were established and cultured as described in Laura Broutier et al*.* [17] (see also Additional file 1: Fig. S1A). In short, liver tissue was resected into small pieces and digested with digestion solution containing 2.5 mg/mL of collagenase D (Sigma-Aldrich, MO, USA) + 0.1 mg/mL of DNAse I (Roche, Almere, the Netherlands) in HBSS (without Ca^+2^ and Mg^+2^). Liver single cells were mixed with 60% Matrigel Matrix Basement Membrane (BD Bioscience, CA, USA) or Cultrex Ultimatrix Reduced Growth Factor Basement Membrane Extract (R&D, MS, USA). After the Matrigel/cell suspension solidified, Expansion Medium (EM) was added. EM was based on AdDMEM/F12 (Thermo Fisher, MA, USA) supplemented with 50% homemade Wnt-3a conditioned medium (medium was collected from LM(TK-) cells, 1 × B27 (Invitrogen) and 1 × N2 (Thermo Fisher), 1.25 mM *N*-acetylcysteine (Sigma-Aldrich), 100 ng/mL FGF10 (PeproTech, CT, USA),10 mM nicotinamide (Sigma-Aldrich), 50 ng/mL HGF (PeproTech), 0.1 µg/mL Rspo-1 (Stemcell, Cologne, Germany) and 50 ng/mL EGF (PeproTech), 100 ng/mL Noggin (R&D), 10 μM Y27632 (Sigma), 0.5 μM A83-02 (Tocris, Bristol, United Kingdom), Forskolin 10 µM (Sigma-Aldrich). Once a week organoids were removed from the Matrigel by mechanical or enzymatic (Triple E, Thermo Fisher) disruption and transferred to fresh Matrigel in a 1:2–1:4 split ratio. Medium was changed every other day. Wnt-3a conditioned medium was prepared described [18]. In brief, normal culture of Wnt-3a cells were in DMEM high glucose (Thermo Fisher) supplemented with 10% FBS (fetal bovine serum, Thermo Fisher) and 1 × Penicillin–Streptomycin (Thermo Fisher) and 125 μg/ml Zeocin (Invitrogen). For the production of WNT3A cells were grown without Zeocin for 2 passages. Wnt3a-conditioned media were collected of second passage, 4 days after reaching 100% confluency, and stored at  − 80 °C until use.

For the treatment of organoids with GDF7, organoids were digested to single cells with Triple E, and seeded in Matrigel for 3 or 4 days and were then treated with rhGDF7 (PeproTech) at concentrations of 10 or 100 ng/ml for 2 days in EM without A83-01 or Noggin, which have been considered as TGFβ pathway inhibitors and therefore may influence GDF7 effect [19].

The HSC cell line LX‐2 (SCC064, Merck, Amsterdam, the Netherlands) described by Xu et al*.* and Smith-Cortinez et al*.* [20, 21] was used in passages 22‐30. DMEM high glucose (Thermo Fisher) supplemented with 10% FBS (fetal bovine serum, Thermo Fisher) and 1 × Penicillin–Streptomycin (Thermo Fisher) was used as normal medium. Prior to HSC activation but after attachment, LX2 cells were starved with no serum‐containing medium for 18 h, Then they were treated with TGFβ1 (Recombinant human transforming growth factor-beta 1, 10 ng/mL, R&D) for 2 days in serum‐free medium (PBS was used as Control). For the treatment of GDF7 on LX2 cells, the cells were first attached and then treated with GDF7 at concentrations of 10 or 100 ng/ml for 2 days in serum‐free medium. After the treatments, RNA was isolated for further analysis.

For the coculture experiment, organoids were mixed with Matrigel and seeded in the 24-well transwell inserts. After 1–2 days, the inserts were put into the wells containing the LX-2 cells that were treated as stated above for 2 days. 250 μL EM was added in the upper layer and 750 μL DMEM was added in the bottom layer. After 2 days, organoids were harvested for RNA isolation and staining.

### Histology, immunohistochemistry, and immunofluorescence

Histology, immunohistochemistry, and immunofluorescence were performed as described previously [20]. In brief, liver tissues were fixed by formalin and then embedded in paraffin. For TMA construction, a hematoxylin and eosin (H&E)-stained section of each liver was used to define representative hepatic lobule regions. Tissue cores with a diameter of 2 mm were randomly obtained from each formalin-fixed paraffin-embedded liver sample and collected into the TMA using a hollow needle (Beecher Instruments, WI, USA). Then glass slides with 4-μm-thick tissue of liver TMA or organoids were deparaffinized in xylene and rehydrated in graded alcohols (100% to 50%). For immunohistochemistry, representative sections of tissue and organoids were treated with antigen retrieval by Sodium citrate (10 mM, pH 6.0) and peroxidase blocking, and then blocked with 2% bovine serum albumin (BSA, Merck) blocking buffer for 30 min. Then sections were incubated with different primary antibodies separately at 4 °C overnight. Subsequently, the sections were washed and re-incubated with secondary antibodies, stained with DAB, dehydrated and mounted in Eukitt (Sigma-Aldrich). Results of immunohistochemistry were quantified by ImageJ software in a blinded manner. Stained slides were scanned with the NanoZoomer 2.0HT slide scanner (Hamamatsu Photonics Europe GmbH, Herrsching am Ammersee, Germany) and analyzed with NanoZoomer Digital pathology viewing software NDP.view2 (Hamamatsu Photonics Europe). A list of antibodies is provided in Additional file 1: Table S1.

For immunofluorescence the deparaffinization, rehydration and antigen retrieval procedures were the same as with immunohistochemistry. Next, sections were incubated with 0.1% Triton X100 (Merck), 2% BSA blocking buffer followed by different primary antibodies. Then, after washing sections were incubated with fluorescent labeled secondary antibodies. Afterward, the sections were sealed with Antifade Mounting Medium with DAPI (Vector laboratories, CA, USA). Finally, sections were visualized by Zeiss 410 inverted laser scan microscope (Leica Microsystems, Wild Heerbrugg, Germany) with 16X or 40X magnification objectives using immersion oil. Organoid quantitative analysis of staining was measured by ImageJ software^18^.

### Presto Blue for cell viability and cytotoxicity

PrestoBlue™ HS Cell Viability assay (Thermo Fisher) was performed following the protocol provided by the supplier. In brief, after the organoids were cultured in 96 wells plates in the absence or presence of rhGDF7 for 2 days, Presto Blue reagent (both diluted in 1:10) was added to the cells for 15 min under the standard culture conditions. After incubation, plates were shaken for 10 min, and the absorbance was measured using a microplate reader at an OD of 450 nm.

### Quantitative reverse transcription-polymerase chain reaction (qRT-PCR)

A qRT-PCR was performed as previously described [22]. Briefly, RNA was isolated from organoids and LX2 using TRIzol® reagent according to the supplier’s instruction (Thermo Fisher Scientific, Bleiswijk, the Netherlands). RNA quality and quantity were tested using a Nanodrop 2000c UVvis spectrophotometer (Thermo Fisher Scientific). cDNA was synthesized using M-MLV reverse transcriptase and random nanomers (Invitrogen). Taqman primers and probes were designed by the software of Primer Express 3.0.1, and sequences are shown in Additional file 1: Table S2. Target genes were amplified using the qPCR core master mix (Eurogentec, Maastricht, the Netherlands) in duplicates on the QuantStudio 3 (Thermo Fisher Scientific). Data were analyzed by using the 2-ΔCt method, and values were normalized to 18 s expression. SDSV2.4.1 (Thermo Fisher Scientific) software was used to analyze the data.

### Liver single-cell sequencing data and analysis

Five raw sequencing data of human liver tissue (GSE115469, GSE124395, GSE130473, GSE136103, and GSE129933) were used in our research [23–27]. GDF7 mRNA, as well as BMPR2, BMPR1A and BMPR1B were analyzed among cells obtained from the fractionation of fresh hepatic tissue in Liver Single Cell Atlas (http://liveratlas-vilarinholab.med.yale.edu/). 15 cell clusters were generated based on expression of the most classic genes and visualized using the t-Distributed Stochastic Neighbor Embedding (t-SNE) technique, which were annotated in different clusters. Targeted mRNA transcriptomics are pooled, and the average normalized protein-coding transcripts per million (pTPM), as well as a normalized expression, are calculated across different clusters [27].

### Statistics

GraphPad Prism version 9.0.0 for Windows was used for statistical analysis and data visualization. The student’s two-way t-test was conducted to assess differences in GDF7 expression between the healthy and fibrotic tissues. Pearson’s Correlation was performed to identify correlations between COL1A1 or ACTA2 and GDF7 expression in liver tissues. Lastly, mean normalized relative expression levels (2-ΔCt) for LGR5, COL1A1 and ACTA2 were compared between different rhGDF7 concentrations using one-way ANOVA or unpaired t-test. Data are presented as mean ± standard error of the mean (SEM). Results were considered statistically different when the *P*-value < 0.05.

## Results

### Hepatic GDF7 expression is enhanced in human liver fibrosis

**Fig. 1:**
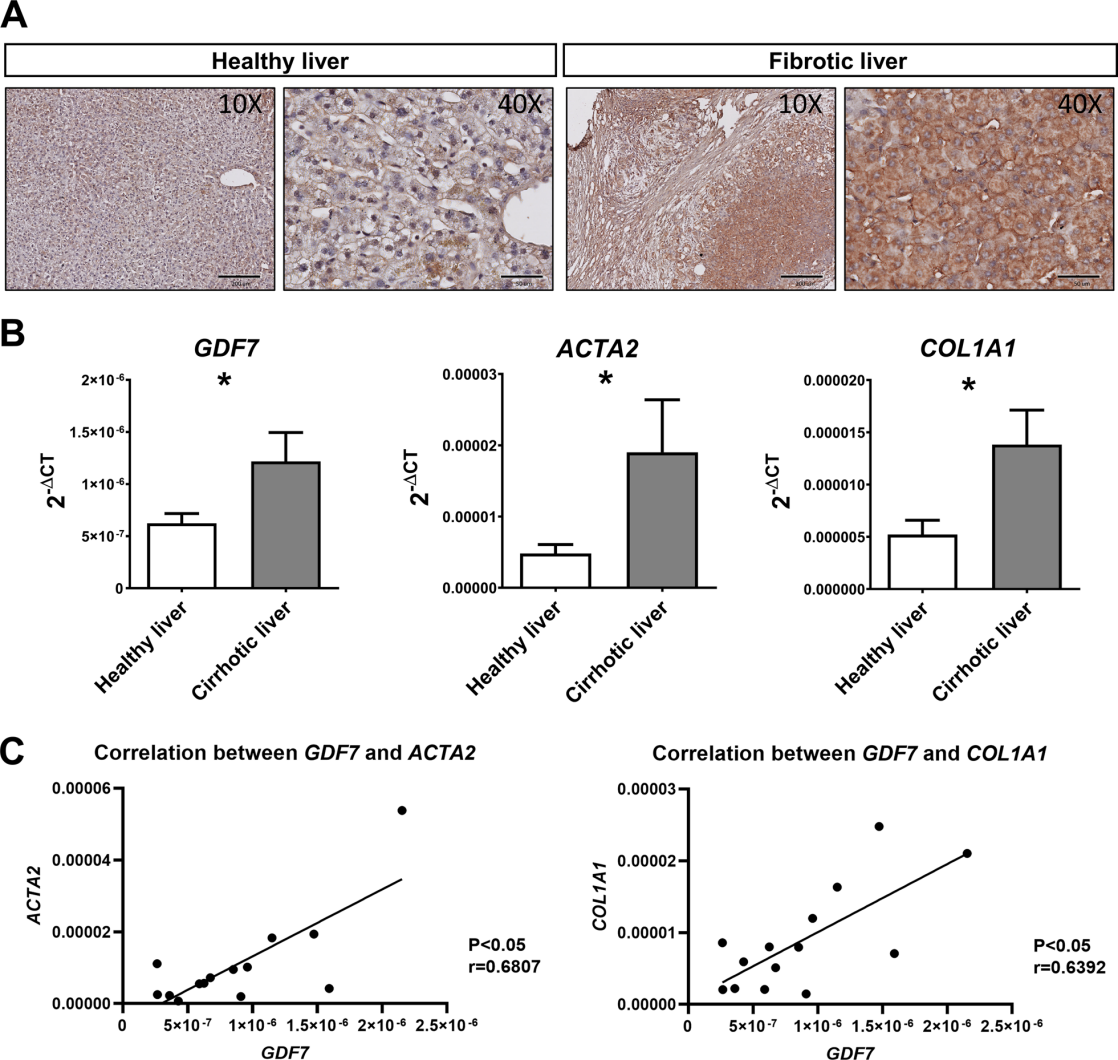
GDF7 is upregulated in liver fibrosis. **A** Immunohistochemical staining of healthy and fibrotic liver tissues, scale bars = 1000 μm (left panel) and 500 μm (right panel). **B** The qPCR analysis for GDF7 expression in fibrotic (red, *n* = 6) and healthy (blue, *n* = 8) liver tissues. Data were presented as mean ± standard error of the mean (SEM) and were considered statistically different when the *p* value (*p*) < 0.05. (**C**) Positive correlation between *GDF7* and *ACTA2* or *COL1A1* expression from liver tissues with fibrosis (*p* < 0.05, *GDF7-ACTA2* r = 0.6807, *GDF7-COL1A1* r = 0.6392; Pearson’s correlation)

Immunohistochemical staining of FFPE tissue of healthy (n = 6) and fibrotic (n = 9) livers revealed an increased presence of GDF7 in fibrotic livers when compared to healthy controls. Notably, GDF7 staining was predominantly confined to the parenchymal regions in fibrotic livers, while fibrotic tissue appeared virtually devoid of GDF7-specific staining (Fig. 1A). In line, *GDF7* mRNA levels were significantly enhanced in human fibrotic livers, along with expression levels of markers of fibrosis, such as *ACTA2* and *Collagen 1A1* (*COL1A1*)[28] (Fig. 1B). In fact, hepatic *GDF7* mRNA levels positively correlated with both *COL1A1* and *ACTA2* mRNA levels in human liver tissue (Fig. 1C).

### GDF7 in human fibrotic livers is mainly detected in hepatocytes and cholangiocytes

**Fig. 2:**
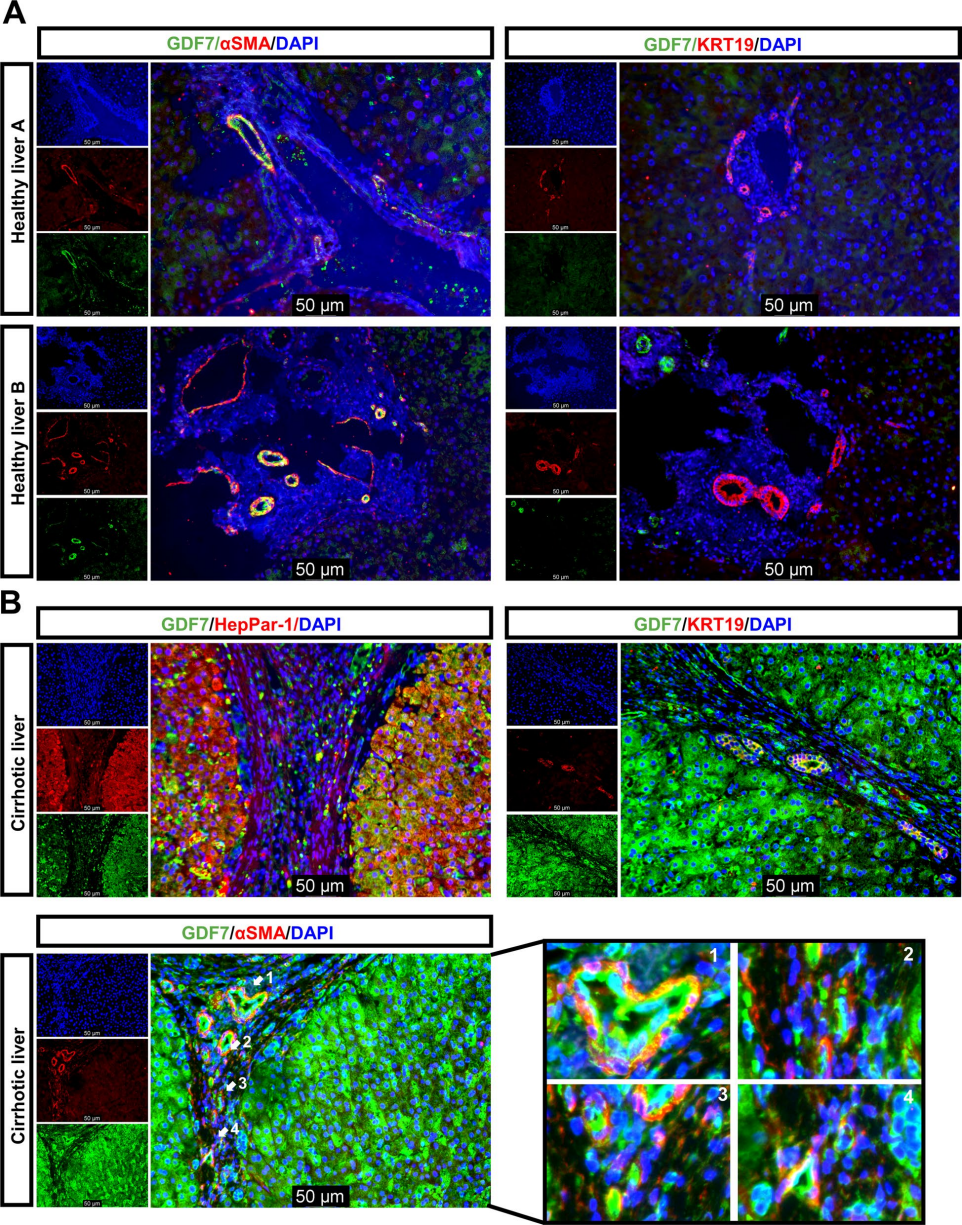
GDF7 was mainly accumulated in hepatocytes and cholangiocytes in liver fibrosis. (**A**) Immunofluorescence co-staining of GDF7 with a-SMA and cholangiocytes marker (KRT19) from two healthy liver tissues. Scale bars, 50 μm. (**B**) Immunofluorescence co-staining of GDF7 with αSMA, cholangiocytes marker (KRT19) and hepatocyte marker (HepPar-1) on fibrotic livers. Scale bars, 50 μm

Co-immunofluorescence staining experiments were performed to locate GDF7 at the cellular level in healthy and fibrotic human liver tissue. In healthy human liver (Fig. 2A), GDF7 staining was predominantly detected in α-SMA-positive vascular structures in the portal area (mainly portal vein blood vessels and hepatic arteries) with some low level GDF7 expression in hepatocytes. GDF7 protein was not detected in KRT19-positive bile ducts in healthy liver tissues. In contrast, a substantial accumulation of GDF7 was observed in hepatocytes (marker: HepPar1) and cholangiocytes (marker: KRT19) in human fibrotic liver tissue, yet it was almost absent in myofibroblasts in the fibrotic bands (marker: α-SMA) (Fig. 2B). Similar to healthy tissue, GDF7 was also detected in aSMA-positive endothelial cells in veins and arteries in fibrotic liver tissue (Fig. 2B, zoom in area 1, 2 and 4 in left panel).

### Possible paracrine and autocrine GDF7 signaling circuits in human liver

**Fig. 3:**
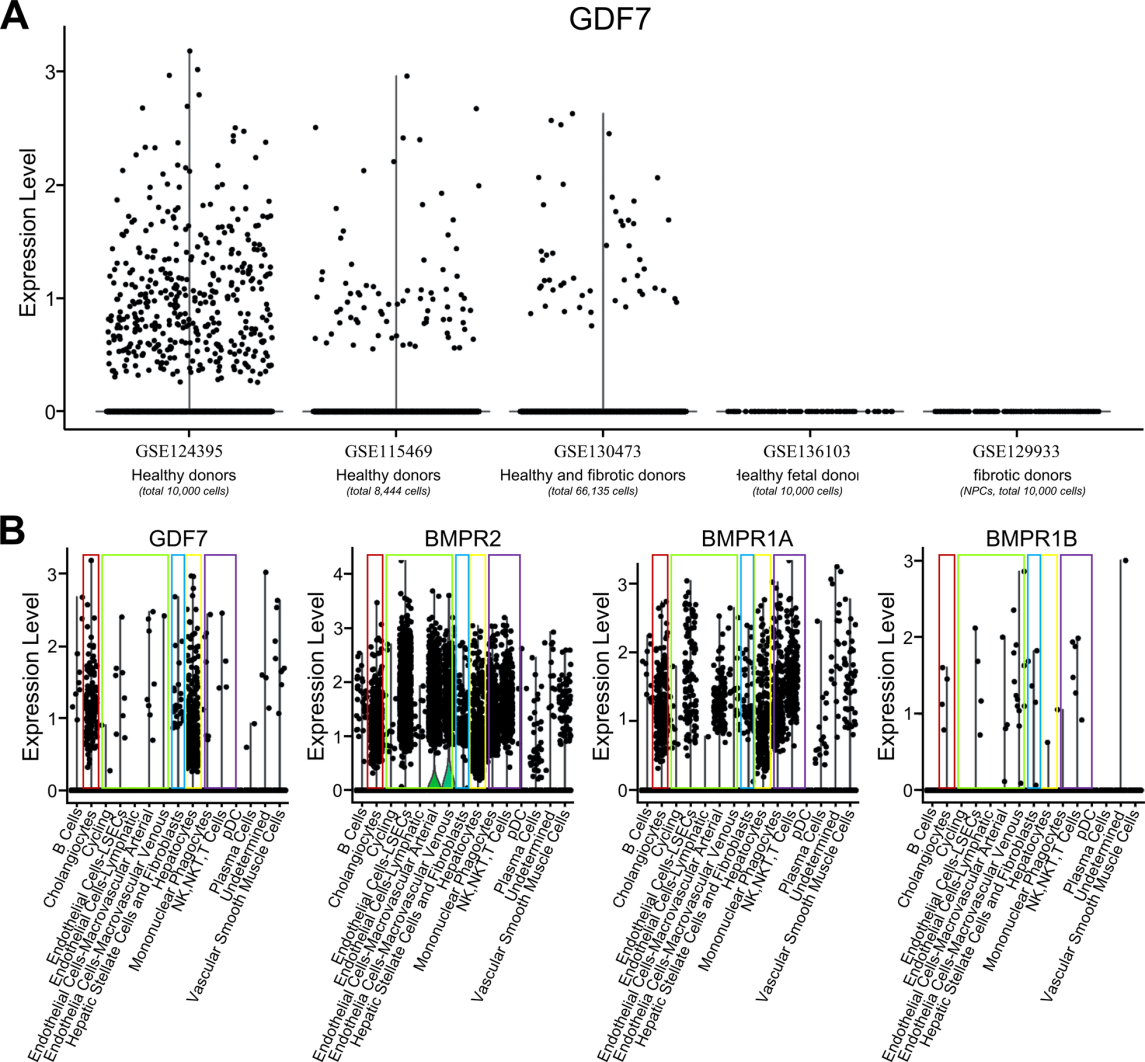
An autocrine signaling circuit of GDF7 in hepatocytes and cholangiocytes occurs in liver fibrosis. (**A**), UMAP plot of cells isolated from human liver tissues, showing different expression level of GDF7 from 5 databases that were identified. (**B**), Relative abundances of *GDF7*, *BMPR2*, *BMPR1A* and *BMPR1B* read counts in single cells were normalized to transcripts in different liver cell types classed by cell type-specific markers. Cholangiocyte types were circled with red line, endothelial types were circled with green line; HSC and fibroblast were circled with blue line, hepatocyte types were circled with yellow line, immune cells were circled with purple line

Several publicly available single cell RNA sequencing databases [23–27] generated from healthy as well as fibrotic human livers were analyzed for *GDF7*-expressing cells, as well as receptors that it may bind to, e.g. bone morphogenetic protein receptor type 2 (BMPR2), BMPR1A (also known as ALK3) and BMPR1B (also known as ALK6) [29] (Fig. 3). Remarkably, *GDF7* mRNA was predominantly detected in hepatocytes and cholangiocytes, and minor amounts in HSC, both in healthy and fibrotic human liver cells, while hardly any expression was detected in endothelial cells, neither venous nor arterial endothelial cells (Fig. 3A, B). This suggests that the parenchymal cells are the main source of GDF7 in liver tissue, which may appear to contradict the immunofluorescence analyses described above (Fig. 2). However, mRNA levels of the GDF7-receptor *BMPR2*, and *BMPR1A* to a lesser extent, were highly enriched in hepatic endothelial cells (indicted in green box in Fig. 3B), which may explain the prominent GDF7 signal on those cells in immunofluorescence microscopy analyses as it may be bound to one of those receptors. Moreover, *BMPR2* and *BMPR1A* mRNA levels were also readily detected in hepatocytes (yellow box), cholangiocytes (red box) and HSC (blue box), as well as in innate and adaptive immune cells (purple box) (Fig. 3B). *BMPR1B* expression was hardly detected in human liver cells. Thus, hepatocytes and cholangiocytes appear the most prominent GDF7-producing cells in healthy and fibrotic liver tissue and may signal via BMPR2A and BMPR1A in an autocrine as well as a paracrine fashion within the liver.

### GDF7 promotes growth of liver tissue-derived organoid but fails to activate HSC

**Fig. 4:**
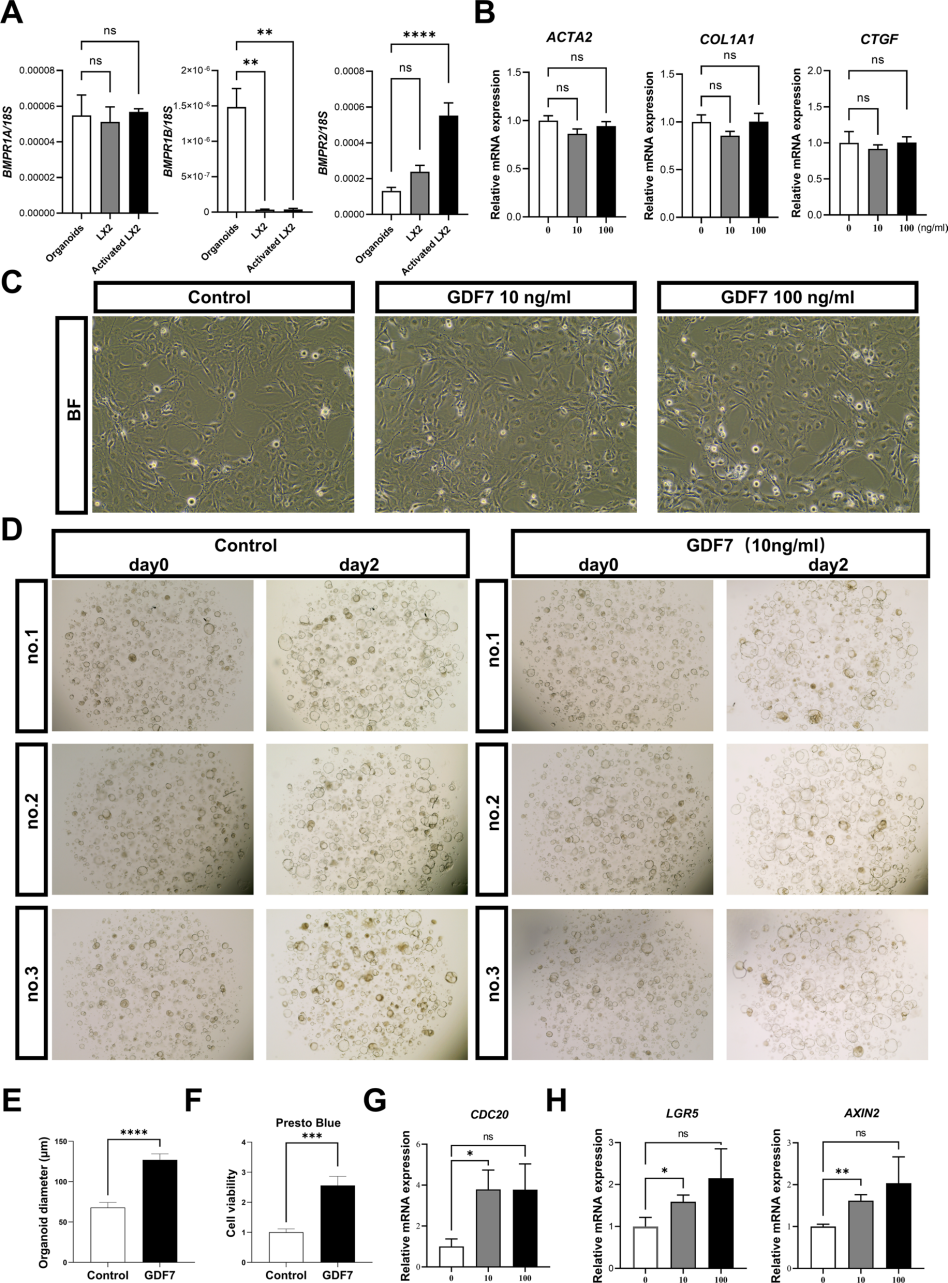
GDF7 promotes growth of liver tissue-derived organoid but fails to activate HSC. (**A**) The qPCR of GDF7 receptors *BMPR1A*, *BMPR1B* and *BMPR2* on liver organoids (*n* = 6), non-activated and activated HSC (*n* = 3). Data represent mean ± SEM. *p < 0.05; ns: no significance, 1-way ANOVA. (**B**) RT-qPCR on LX2 cells treated with rhGDF7 for the HSC activation markers *COL1A1* and *ACTA2*. Experiments were repeated at least 3 times. Data represent mean ± SEM. *p < 0.05; ns: no significance, 1-way ANOVA. (**C**) Representative images of HSC LX2 cell line treated with GDF7 at the concentration of 10 or 100 ng/ml for 2 days, Bar = 1000 µm. (**D**) Representative images of liver organoids treated with GDF7 at the concentration of 10 ng/ml for 2 days, and the quantification of organoid size (**E**), Bar = 1000 µm. (**F**) Cell viability measurement was performed using Presto Blue reagent. Relative cell viability was calculated by normalizing to 0 ng/ml after 2 days of 10 ng/ml rhGDF7 incubation (*n* = 5) (**G**) RT-qPCR on liver organoids that treated with GDF on cell proliferation markers *CDC20*. (**H**) RT-qPCR on liver organoids that treated with GDF on stem cell markers *LGR5* and *AXIN2*. Experiments were repeated at least 3 times. Data represent mean ± SEM. *p < 0.05; *ns* no significance, 1-way ANOVA

Given the positive correlation between the expression of hepatic GDF7 and markers of fibrosis, and its potential for paracrine and autocrine signaling within the liver, we next used and analyzed the effect of recombinant human (rh)GDF7 on LX-2 HSC and tissue-derived human liver organoids. The procedure of obtaining liver human adult LGR5-positive progenitor cell-derived organoid is shown in Additional file 1: Fig. S1A. Similar to the scRNAseq data, *BMPR2* and *BMPR1A* mRNA levels were readily detected in both liver organoids and LX-2 cells also after treatment of the latter with TGFβ1 (Fig. 4A). *BMPR1B* mRNA levels were much lower in LX-2 cells compared to liver organoids, but for both cell types the CT values suggested only marginal expression of this receptor when compared to *BMPR2* and *BMPR1A*. Overall, these data suggest that these in vitro-cultured cells may be used to model an in vivo response to GDF7. RhGDF7 (0–100 ng/ml for 48 h) exposure did not affect cell growth and morphology of LX-2 cells (Fig. 4C), nor did it change mRNA levels of HSCs activation markers *ACTA2*, *COL1A1* or *CTGF* [28, 30] (Fig. 4B). In contrast, rhGDF7 (10 ng/ml) promoted liver organoid growth compared to non-treated controls (Fig. 4D, with quantification in 4E), which was further validated by the Presto Blue cell viability assay (Fig. 4F). Furthermore, rhGDF also increased mRNA levels of the proliferation marker *CDC20* (Fig. 4G). In addition, rhGDF7 dose-dependently enhanced gene expression of the stem cell markers *LGR5* and *AXIN2* (Fig. 4H). These data show that GDF7 has the potential to promote liver progenitor cell proliferation, more so than affecting HSC activation.

### Activated HSC induce GDF7 expression in human liver organoids

**Fig. 5:**
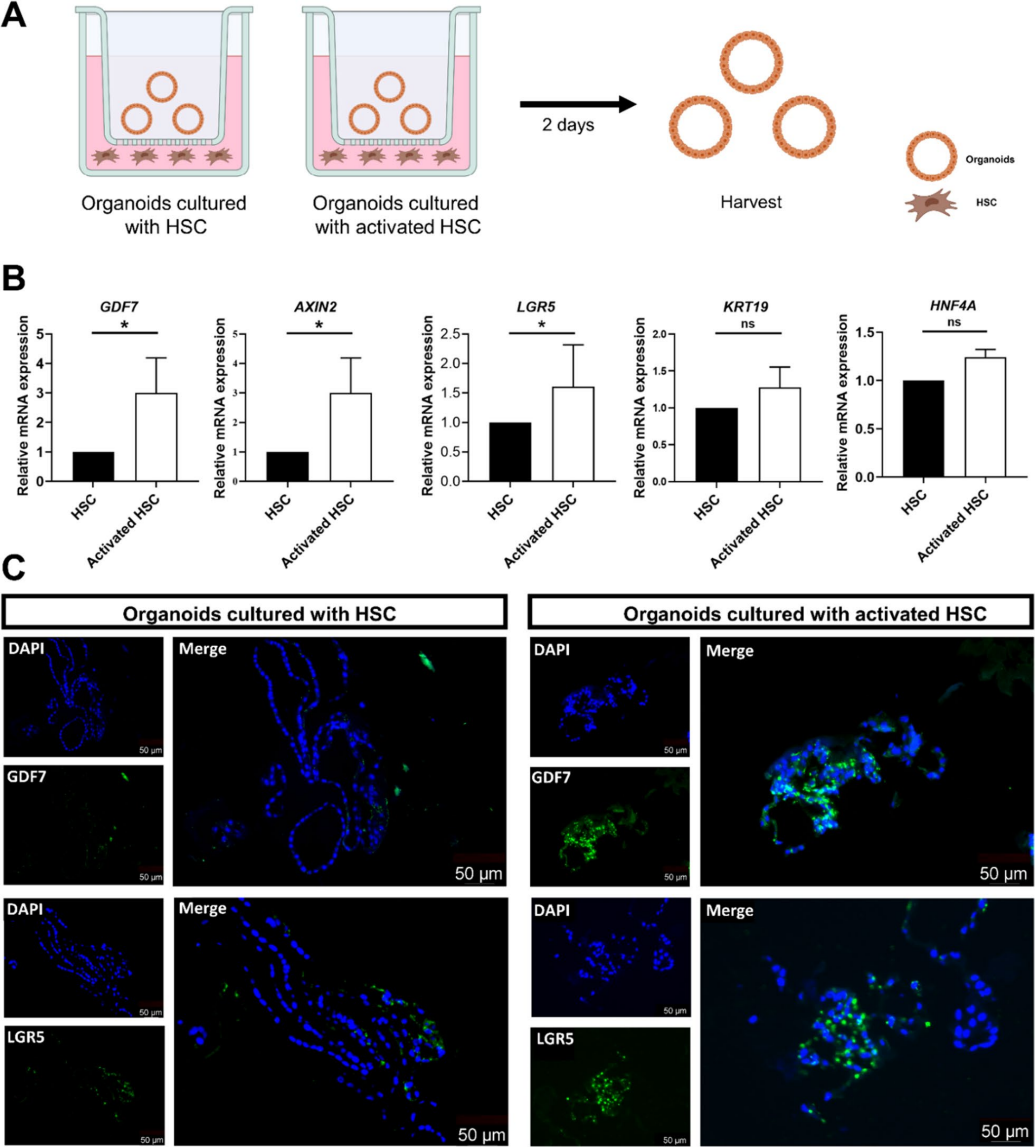
Activated HSC induce GDF7 expression in human liver organoids. (**A**) Schematic overview of experimental design of human liver organoids cocultured with human activated HSC LX2 cell line using transwell system. (**B**) Anti-GDF7 and LGR5 immunofluorescence of GDF7 and LGR5 staining on a series sections of liver organoids co-cultured with non-activated and activated HSC LX2 cells, Data represent mean ± SEM. *p < 0.05; **p < 0.01 as compared to control, unpaired Student’s *t*-test. (**C**). Immunofluorescence of GDF7 and LGR5 staining on a series sections of liver organoids co-cultured with non-activated and activated HSC LX2 cells, Bar = 50 μm

**Fig. 6:**
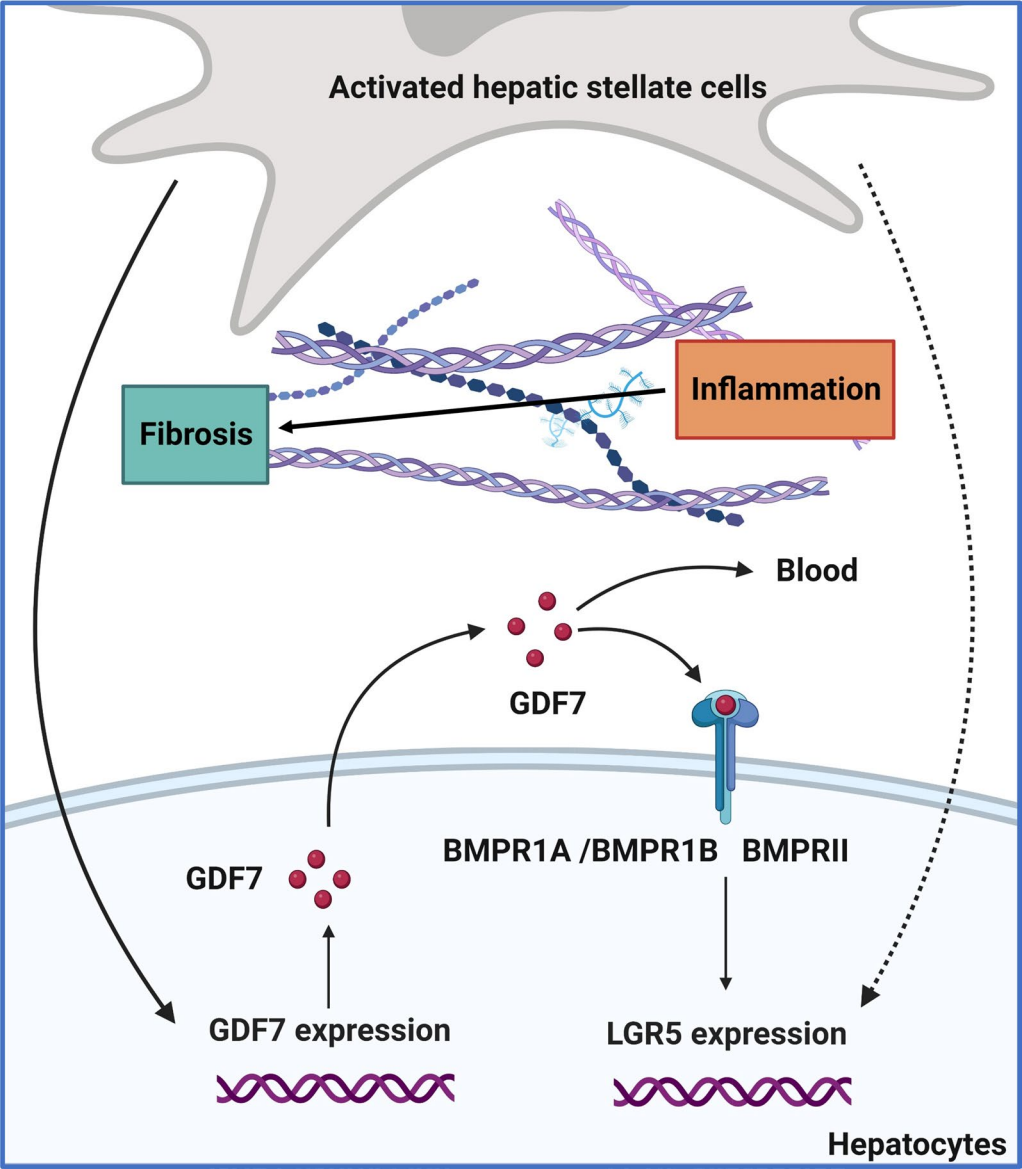
The enhanced autocrine GDF7 signaling circuit in hepatocytes in fibrotic microenvironment promotes LGR5 expression during liver disease. Chronic liver damage leads to chronic liver inflammation, and this causes the (over)activation of hepatic stellate cells that lead to fibrosis. Inflammation and hepatic fibrosis therefore co-exist in chronic liver diseases [32]. GDF7 is an autocrine regulator of liver regeneration through its control of LGR5 liver progenitor cells (LPC). GDF7 is produced by hepatocytes and can bind to its type I receptors BMPR1A and BMPR1B that are highly expressed by hepatocytes as heterocomplexes with the type II receptors BMPR2. GDF7 acts in an autocrine manner on hepatocytes to promote LGR5 LPC proliferation in liver fibrosis, in which HSC are activated, synthesize collagen fibers, and become pro-fibrotic, and inflammatory cytokines are simultaneously recruited into the fibrotic areas

In order to identify mechanisms responsible for the enhanced expression of GDF7 in hepatocytes in fibrotic liver tissue, we mimicked the fibrotic microenvironment in vitro by coculturing liver organoids with activated HSC LX2 cells in a transwell system (Fig. 5A). TGFβ1-activated LX-2 cells significantly enhanced *GDF7* expression in co-cultured human liver organoids, when compared to control HSC (Fig. 5B). In line with our earlier study (data not published), TGFβ1-activated LX-2 also increased gene expression of progenitor markers *LGR5* and *AXIN2*, while it did not affect expression of hepatocyte (*HNF4A*) and cholangiocyte (*KRT19*) marker genes [31]. Immunofluorescence microscopy confirmed that TGFβ1-activated LX2 cells induced GDF7 protein expression in cocultured human liver organoids, when compared to coculture with control-grown LX-2 cells (Fig. 5C). In addition, the LGR5 staining of the same organoids in a serial section revealed that TGFβ1-activated LX-2 cells also enhanced LGR5 protein expression in the liver organoids, as shown previously (data not published). Collectively, these data indicate that activated HSC induce GDF7 in liver parenchymal progenitor cells, which in turn may promote liver regeneration via autocrine signaling (Fig. 6).

## Discussion

This study shows that hepatic GDF7 expression positively correlates with the progression of human liver fibrosis. Activated HSC, which do not express GDF7 themselves, induce expression of GDF7 in parenchymal cells, e.g., hepatocytes and cholangiocytes, which promotes proliferation and progenitor marker expression in liver organoids, revealing a novel potential function for GDF7 in liver regeneration during liver fibrosis.

GDF7 is one of over 30 members of the large TGFβ superfamily. TGFβ signaling pathways play essential roles in the regulation of different cellular processes that are essential for maintaining tissue and organ homeostasis, including cell proliferation, differentiation, migration and cell death [33]. In the liver, TGFβ signaling is involved in all stages of disease progression, from liver injury through inflammation and fibrosis, to cirrhosis and cancer. TGFβ1 exhibits anti-cytostatic and anti-apoptotic effects in hepatocytes, which promotes liver differentiation during embryogenesis and physiological liver regeneration. However, chronic liver damage leads to persistent high levels of TGFβ1, which induces the transactivation of hepatic stellate cells that produce excessive amounts of extracellular matrix proteins giving rise to liver fibrosis that may progress to cirrhosis [33]. In contrast to TGFβ1, the role of BMPs/GDFs in the regulation of liver regeneration and fibrosis is complex. Previous studies have reported upregulation of BMPs/GDFs, while they exhibited different roles in liver injury or fibrosis [34–36]. Some BMPs/GDFs have been identified as promoters of liver fibrosis. For instance, BMP4 exerts a pro-fibrotic effect by promoting HSC activation through stimulation of ERK1/2 and Smad1 in the cholestatic rat model of bile duct ligation [37]. In addition, BMP9 knock-out in mice significantly inhibited HSC activation and reduced fibrosis in carbon tetrachloride (CCl_4_)-induced liver injury. However, there are also some BMPs/GDFs that effectively attenuate HSC activation and subsequently liver fibrosis. Chung et al. showed that BMP2 was downregulated in human liver fibrosis and in CCl_4_-induced liver fibrosis in mice, while overexpression of BMP2 remarkably mitigated HSC activation [38]. Similar findings were reported for BMP7, overexpression of which decreased the expression of collagen in HSC and suppressed fibrosis via the upregulation of Id proteins in thioacetamide-induced liver fibrosis in mice [39]. Additionally, loss of BMP6 was found to exacerbate liver fibrosis in a mouse model of non-alcoholic fatty liver disease, while recombinant BMP6 inhibited HSC activation and reduced profibrogenic and inflammatory gene expression in activated HSC in vitro [40]. Our data show that GDF7 expression is strongly upregulated in human fibrotic livers, which aligns with increased *GDF7* gene expression in blood cells in patients with liver fibrosis reported by others [16]. However, we did not observe a direct fibrogenic effect of GDF7 treatment of human LX2-HSC in vitro, as it did not affect gene expression of *COL1A1*, *ACTA2* and *CTGF*. Apart from directly regulating HSC activation, some BMPs/GDFs may ameliorate hepatic fibrosis by increasing expansion of liver progenitor cells (LPC) that aid in liver regeneration by differentiation to cholangiocytes or hepatocytes [41]. A contributor to tissue regeneration during chronic liver damage is the expansion of LGR5 + LPC. LGR5 + cells are a population of stem cells found in multiple tissues, yet are very rare in the healthy mouse liver [18, 42]. Factors like HGF and/or Rspo1 have been shown to promote expansion of these LGR5 + cells and attenuate liver fibrosis in mice [34]. A similar effect was reported for GDF11, e.g., GDF11 is upregulated in human fibrotic liver and ameliorated liver fibrosis in CCl_4_-treated mice by promoting LGR5 + LPC expansion [35]. In line, we found that GDF7 induces the expression of *LGR5*, as well as the expression of another progenitor cell marker *AXIN2*, in human liver organoids. Moreover, it induced expansion of liver organoids, suggesting a similar mechanism as observed for GDF11.

BMPs and GDFs activate Smad-dependent (canonical) and several Smad-independent (non-canonical) signaling pathways to regulate gene transcription. The initiation of the signal transduction cascade occurs when BMPs/GDFs bind to cell surface receptors and form a heterotetrameric complex that consists of two dimers of type I and type II serine/threonine kinase receptor [43, 44]. The type I receptor contains two additional motifs, a glycine/serine-rich region preceding the kinase domain (GS-box) and a short region of eight amino acids, termed L45 loop, within its kinase domain. The type II receptor kinase is constitutively active. In addition, the specificity of the intracellular signals is mainly determined by type I receptors. There are five known BMP type I receptors (BMPR1) that are available for BMPs/GDFs [44]. the activin receptor-like kinase 1 (ACVRL1 or ALK1); type 1A activin receptor (ActR-1A or ALK2); BMP receptor type 1A (BMPR1A, also known as ALK3); the activin receptor type-1B (ACVR1B or ALK-4) and type 1B BMP receptor (BMPR1B or ALK6) [44]. BMPR1A, BMPR1B and BMPRII have been demonstrated as receptors of GDF7 [29, 45]. Both BMPR1A and BMPRII are expressed by hepatocytes, cholangiocytes and HSC in vivo, as well as in organoids and LX-2 cells in vitro suggesting potential responsiveness to GDF7. Still, GDF7 only affected growth and gene expression in human liver organoids and not in LX2 cells, at least not changing the expression of markers of fibrosis in the latter. It remains to be determined whether BMPR1A and/or BMPRII protein differ between these cell types or whether specific components of downstream pathways are differentially present in these cell types. Interestingly though, activated HSC-LX2 cells that do not express GDF7 themselves, induced both gene and protein levels of GDF7 in human liver organoids. This indicates that factors from HSC control GDF7 expression in liver parenchymal cells and may aid in liver regeneration by promoting LGR5 + cell expansion. Moreover, HSC-induced expression of GDF7 in hepatocytes and cholangiocytes likely ends up in circulation, and together with enhanced GDF7 expression by blood cells [16] may serve as a marker of liver fibrosis. Since GDF7 does promote proliferation of parenchymal cells (as in organoids) and does not active a pro-fibrotic phenotype in HSC, it may very well have therapeutic applications, especially promoting liver regeneration in fibrotic liver disease. For example, applying recombination human GDF7 or activation of GDF7 receptors and downstream pathways are potential therapeutic approaches to promote liver regeneration in patients with liver fibrosis. Future research should establish whether such approach indeed improves liver function without increasing the risk for liver cancer.

Besides its effect on hepatocytes and cholangiocytes, GDF7 likely also affects endothelial cells as significant GDF7-specific staining was observed on vascular structures in healthy and fibrotic liver tissue. Indeed, BMPs/GDFs have been shown to influence endothelial cell behavior and regulate blood vessel formation and vascular homeostasis [46]. Moreover, BMP9 was found to protect against hepatic fibrosis by controlling liver sinusoidal endothelial cell fenestration [47]. Potential effects of GDF7 on other cell types in liver fibrosis remain to be determined in future studies.

## Conclusion

Our results show that the autocrine signaling of GDF7 enhanced by activated HSC in parenchymal liver cells promote expansion of liver progenitor cells to aid in liver regeneration in fibrotic liver disease, which suggests a potential pro-regenerative function that can be utilized for amelioration of hepatic fibrosis.

### Supplementary Information

**Additional file 1: Figure S1.** The process of establishment of human liver organoids from healthy liver tissues, and liver organoids were identified as LGR5 positive.** Table S1.** Table S1. The list of antibodies.** Table S2.** The list of qPCR primers.

## Data Availability

All data generated or analyzed during this study are included in this published article.

## Abbreviations

BMP: Bone morphogenic proteins
BMPR: Bone morphogenetic protein receptor
COL1A1: Collagen, type I alpha 1
DAPI: 4’,6-Diamidino-2-phenylindole
ECM: Extracellular matrix
HSC: Hepatic stellate cells
GDF: Growth differentiation factor
Hep Par-1: Hepatocyte paraffin 1
KRT19: Cytokeratin 19
LGR5: Leucine-rich-repeat-containing G protein-coupled receptor 5
LPC: Liver progenitor cells
MFs: Myofibroblasts
TMA: Tissue microarray
TGFβ1: Transforming growth factor-beta 1
