# Supplementary material for: Growth differentiation factor 7 autocrine signaling promotes hepatic progenitor cell expansion in liver fibrosis
Source: Stem Cell Res Ther. 2023 Oct 5;14:288. doi: 10.1186/s13287-023-03493-3 (PMC10557292; doi:10.1186/s13287-023-03493-3)
Supplement: Supplementary file 1 — Additional file 1: Figure S1. The process of establishment of human liver organoids from healthy liver tissues, and liver organoids were identified as LGR5 positive. Table S1. Table S1. The list of antibodies. Table S2. The list of qPCR primers. [file 13287_2023_3493_MOESM1_ESM.docx]

**SUPPLEMENTARY MATERIAL**

**
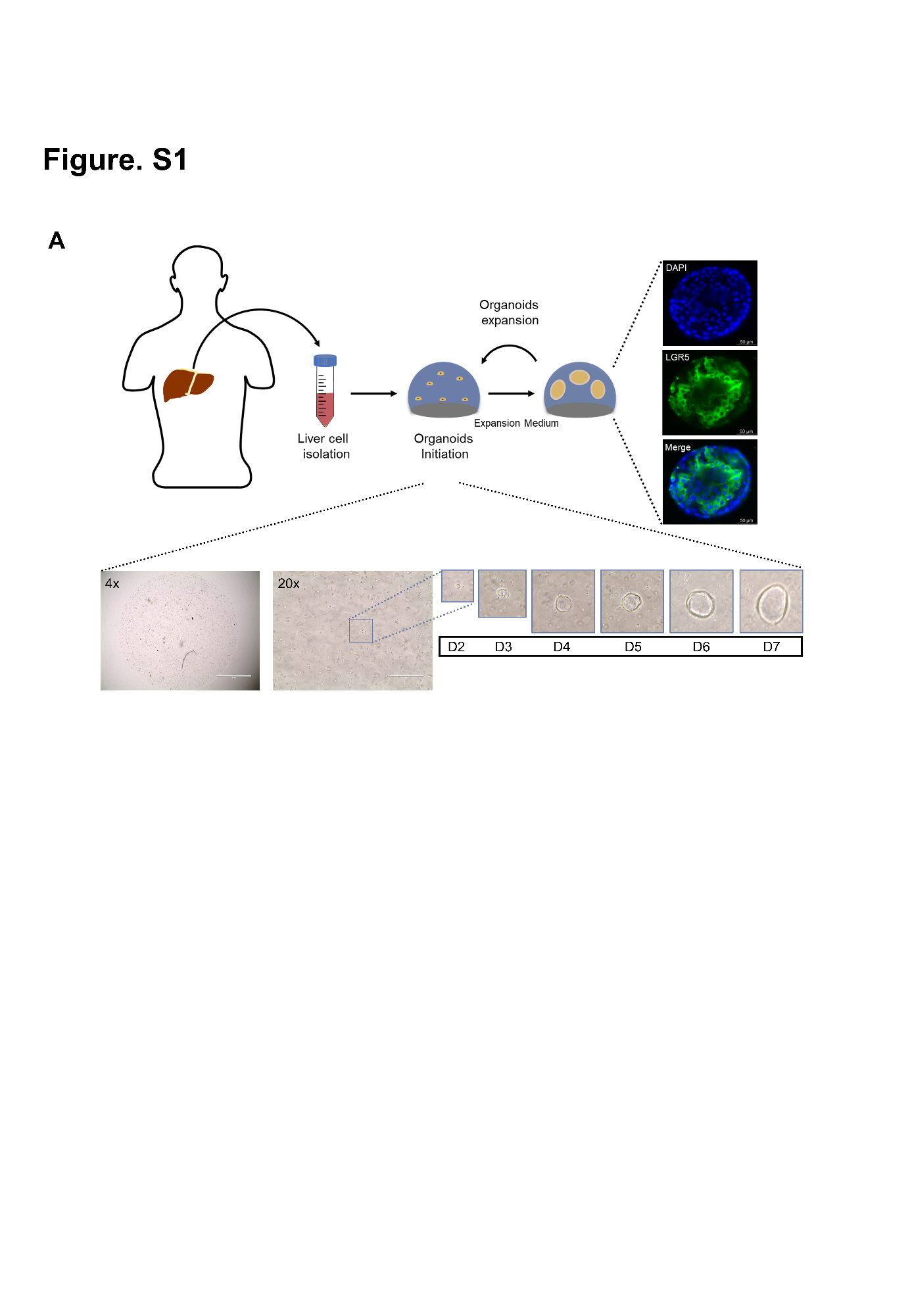
**

**Figure S1.**  The process of establishment of human liver organoids from healthy liver tissues, and liver organoids were identified as LGR5 positive.

**Table S1. The list of antibodies.**

**
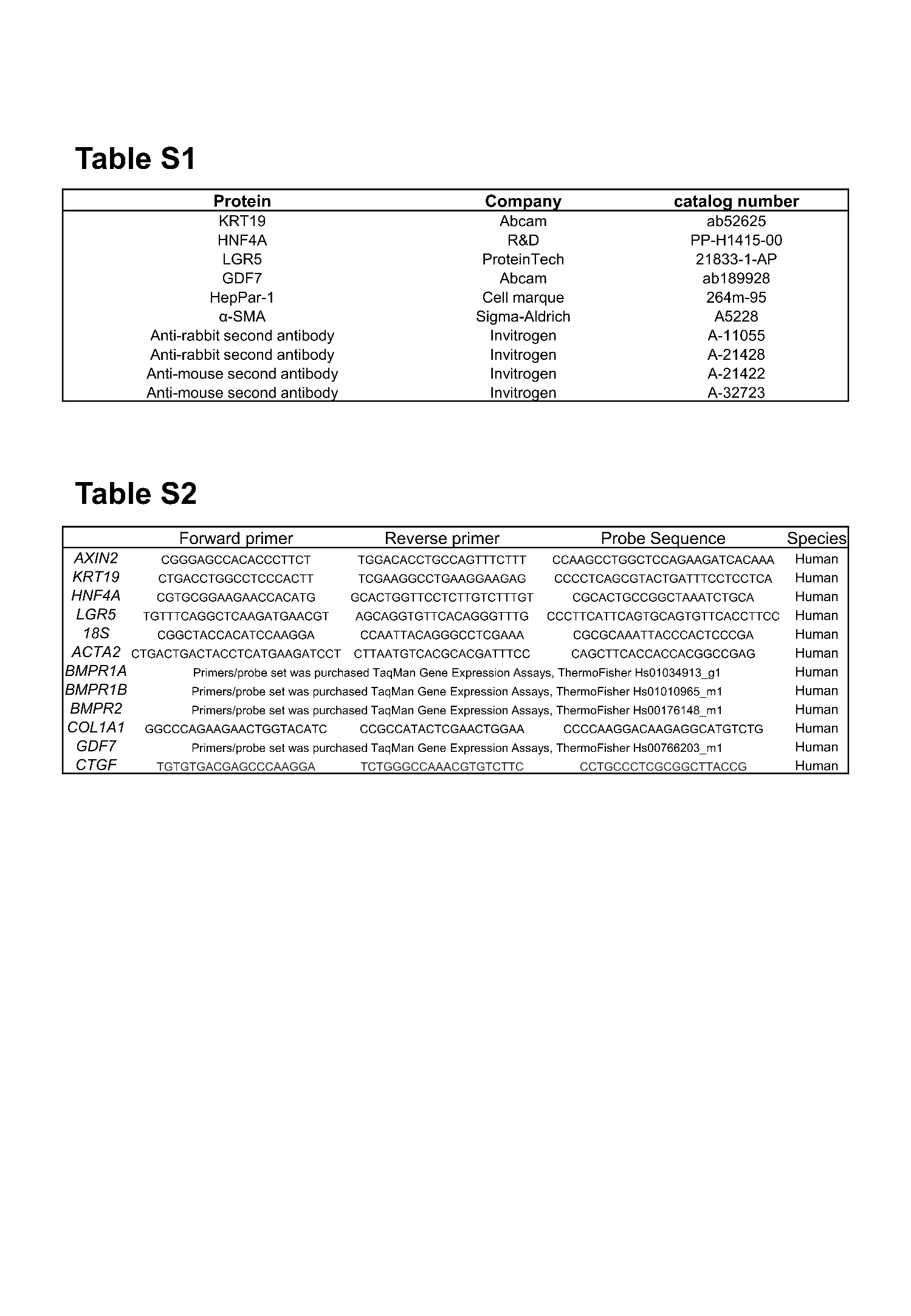
**

**Table S2. The list of qPCR primers.**

**
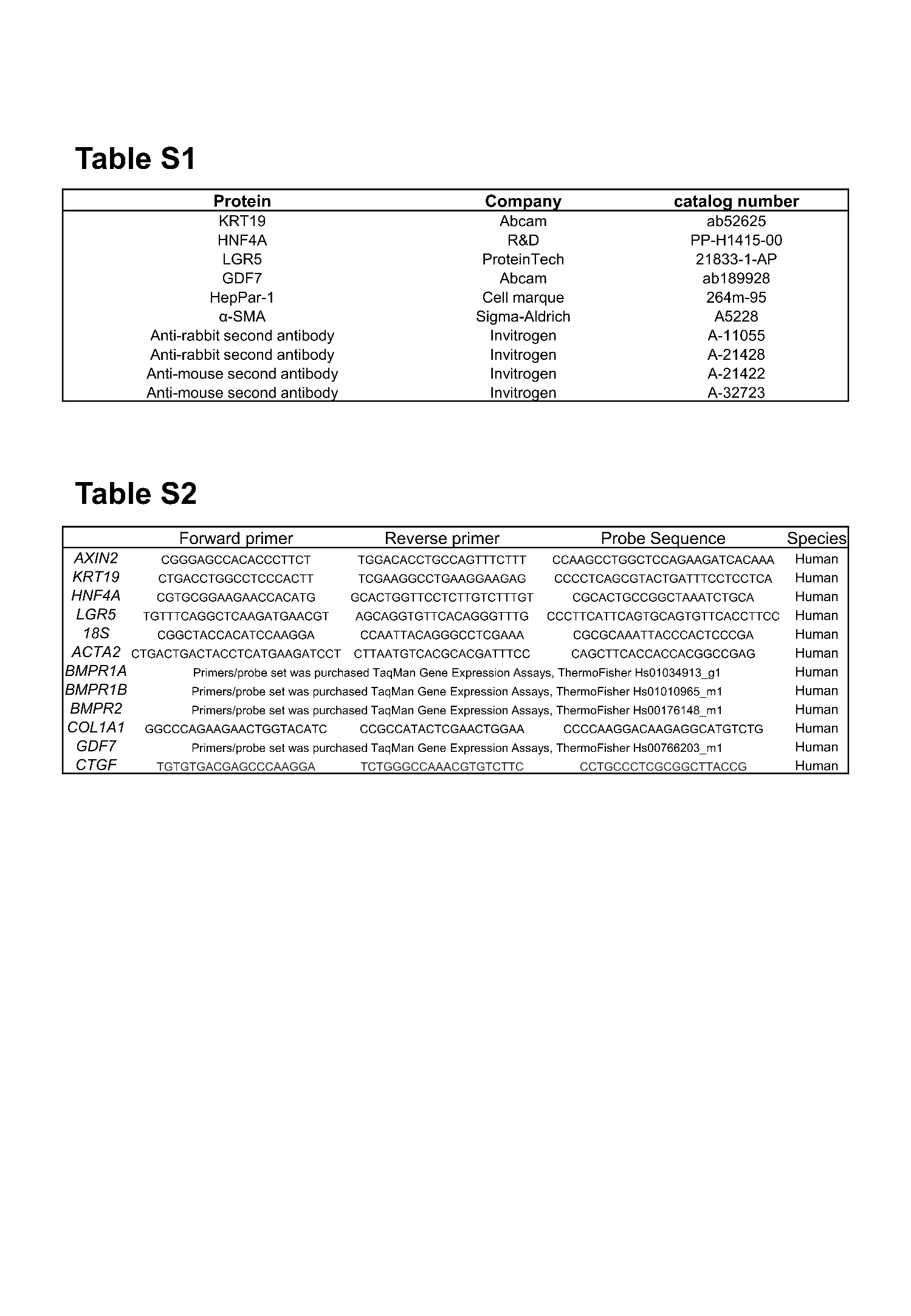
**
